# Foraging strategies, craniodental traits, and interaction in the bite force of Neotropical frugivorous bats (Phyllostomidae: Stenodermatinae)

**DOI:** 10.1002/ece3.8014

**Published:** 2021-09-23

**Authors:** Leidy Viviana García‐Herrera, Leidy Azucena Ramírez‐Fráncel, Giovany Guevara, Gladys Reinoso‐Flórez, Alfonso Sánchez‐Hernández, Burton K. Lim, Sergio Losada‐Prado

**Affiliations:** ^1^ Programa de Doctorado en Ciencias Biológicas & Grupo de Investigación en Zoología (GIZ) Facultad de Ciencias Universidad del Tolima Ibagué Colombia; ^2^ Departamento de Biología & Grupo de Investigación en Zoología (GIZ) Facultad de Ciencias Universidad del Tolima Ibagué Colombia; ^3^ Departamento de Matemáticas y Estadística Facultad de Ciencias Universidad del Tolima Ibagué Colombia; ^4^ Department of Natural History Royal Ontario Museum Toronto ON Canada

**Keywords:** ecomorphology, frugivores, morphometry, skull, Stenodermatinae, trophic performance

## Abstract

1. Bats in the family Phyllostomidae exhibit great diversity in skull size and morphology that reflects the degree of resource division and ecological overlap in the group. In particular, the subfamily Stenodermatinae has high morphological diversification associated with cranial and mandibular traits that are associated with the ability to consume the full range of available fruits (soft and hard).
2. We analyzed craniodental traits and their relationship to the bite force in 343 specimens distributed in seven species of stenodermatine bats with two foraging strategies: nomadic and sedentary frugivory. We evaluated 19 traits related to feeding and bite force in live animals by correcting bite force with body size.
3. We used a generalized linear model (GLM) and post hoc tests to determine possible relationships and differences between cranial traits, species, and sex. We also used Blomberg's K to measure the phylogenetic signal and phylogenetic generalized least‐squares (PGLS) to ensure the phylogenetic independence of the traits.
4. We found that smaller nomadic species, *A. anderseni* and *A. phaeotis* , have a similar bite force to the large species *A. planirostris* and *A. lituratus*; furthermore, *P. helleri* registered a bite force similar to that of the sedentary bat, *S. giannae*. Our study determined that all the features of the mandible and most of the traits of the skull have a low phylogenetic signal. Through the PGLS, we found that the diet and several cranial features (mandibular toothrow length, dentary length, braincase breadth, mastoid breadth, greatest length of skull, condylo‐incisive length, and condylo‐canine length) determined bite force performance among Stenodermatiane.
5. Our results reinforce that skull size is a determining factor in the bite force, but also emphasize the importance of its relationships with morphology, ecology, and phylogeny of the species, which gives us a better understanding of the evolutionary adaptions of this highly diverse Neotropical bat group.

## 1 INTRODUCTION

The skull of vertebrates is a complex structure that is closely associated with resource collection, food processing, and behavior of the species (Bels & Herrel, 2019). Examining the patterns and mechanisms that lead to cranial variation, including size and shape, enables an understanding of the morphology, ecology, and general fitness of animals (Santana et al., 2010). Variation in cranial morphology of bats is attributed to evolutionary processes of ecological specialization, which results in an ecomorphological niche division between similar species (Santana et al., 2012). This variation responds mainly to functional requirements related to nutritional performance and the sensory system (Thiagavel et al., 2018). Among the bats of the New World, rostral length is the main morphological feature that has allowed morphological differentiation, diversity of food, and ecological niches (Hedrick et al., 2020; Shi et al., 2021).

Among chiropterans, New World leaf‐nosed bats (Phyllostomidae) represents one of the largest and most morphologically diverse mammal families (Rossoni et al., 2017). Phyllostomids have the highest diversity of bats in the Neotropics with more than 70 species that can be found in sympatry (Giannini & Kalko, 2004; Reid et al., 2015). Ecological diversification in Phyllostomidae is related to bite performance and mechanical demands of different diets, including frugivorous, insectivores, nectarivores, carnivores, and sanguinivores (Dumont, 2007; Manhães et al., 2017; Nogueira et al., 2009). Dietary differences require specific mechanical modifications, including variation in the rostral length and height of the skull (Santana et al., 2010). However, there is a lack of understanding about the patterns in the variation of shape and performance (López‐Aguirre & Pérez‐Torres, 2015). The remarkable specializations seen in these bats provide a unique opportunity for studying the relationship between cranial morphology, feeding performance, foraging strategy, and dietary ecology (Aguirre et al., 2002; Rossoni et al., 2017; Soriano, 2000).

Within Phyllostomidae, the Stenodermatinae subfamily contain >43% of all described New World leaf‐nosed bat species (Shipley & Twining, 2020). In central Colombia, Department of Tolima, this subfamily represents 13% of species diversity (see García‐Herrera et al., 2019a). Frugivorous bats provide an excellent model to study the relationship between craniodental morphology and bite force because they have different foraging strategies (Soriano, 2000) and consume both hard and soft fruits, which are the result of adaptive pressures related to mandibular morphology (see Murillo‐García & De la Vega, 2018).

Although various studies have addressed the relationships of craniodental morphology, bite force, and diet (e.g., Aguirre et al., 2002; Dumont et al., 2009; Santana et al., 2012; Santana & Miller, 2016), the evaluation of these variables has been carried out individually (Shi et al., 2020); there are existing information gaps that make it difficult for us to understand the morphological features associated with the diet of fruit bats. According to Soriano (2000), the Stenodermatinae subfamily are the only ones that present two foraging strategies for fruit consumption. Nomadic bats present a wide range of home, presenting preferential consumption of figs (*Ficus*), hard fruits, while the members of the sedentary strategy (only the genus *Sturnira*) prefer soft fruits (*Solanum*), reflecting specialization toward a specific fruit or group of fruits (Santana et al., 2010, 2012).

The objectives of our study were to identify the functional traits associated with bite force and the effects of foraging strategies in seven representative species of Stenodermatinae occurring in Colombia to address trophic ecology in a phylogenetic context. We hypothesize that nomadic frugivorous species will have a greater biomechanical advantage of stronger bite force irrespective of size in relation to sedentary frugivorous bats, because they have to carry their food further to their roosts. While the sedentary diet has fixed feeding points.

## 2 MATERIALS AND METHODS

### 2.1 Field collection and laboratory analysis

**FIGURE 1:**
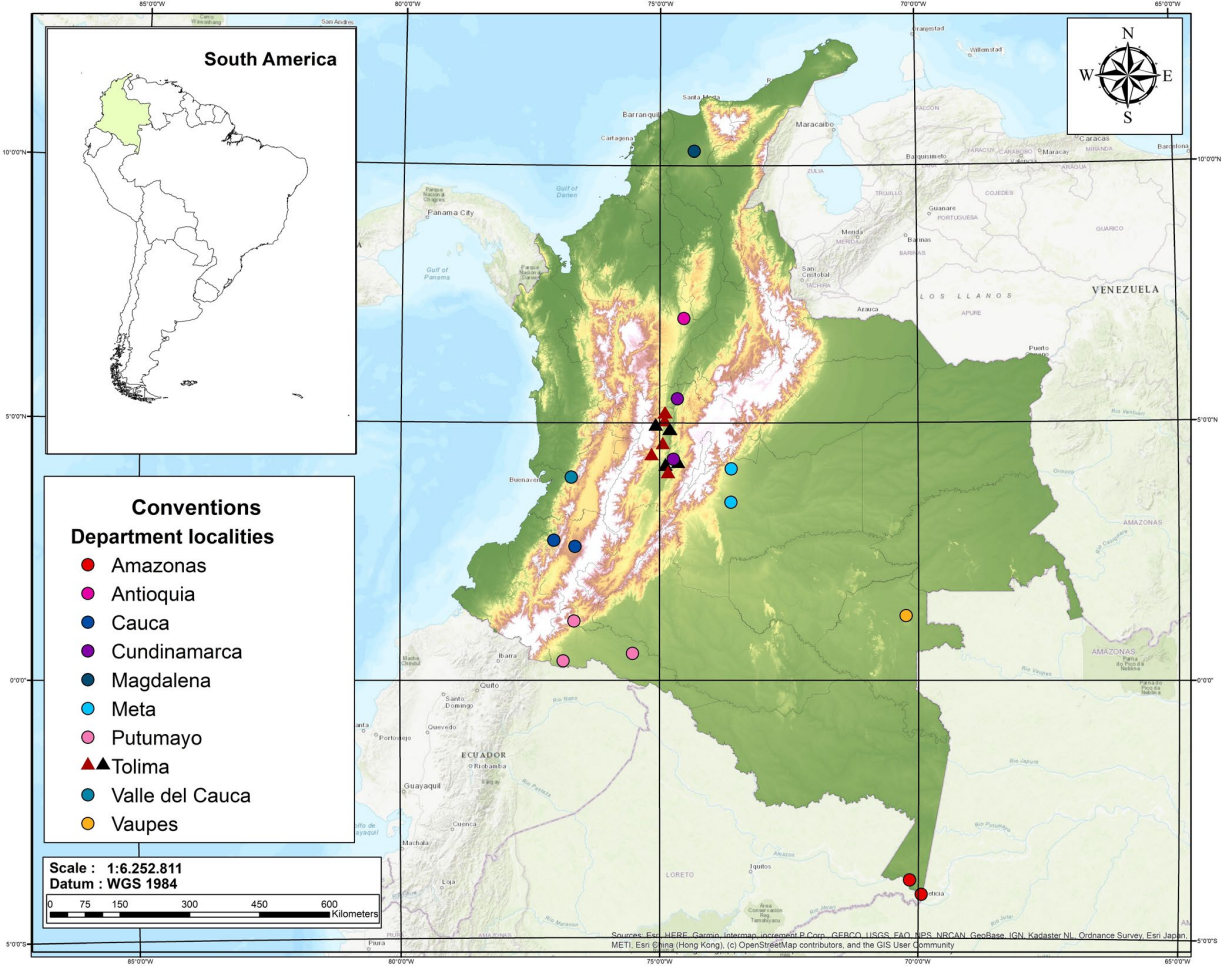
Geographic distribution of the locations in Colombia of the stenodermatine bat samples used for the biometric analyses

We followed two procedures for collecting data. The first involved fieldwork in selected areas of the Colombian tropical dry forest (TDF) in the Department of Tolima (Figure 1; Appendix A) from February 2019 to January 2020. Conventional survey methodology was used, including mist nets placed along trails within forest areas, at the edge of forest remnants, and near waterbodies. Each sampling night consisted of four standard‐size mist nets (12 × 2.5 m) in the forest understory, eight nets (6 × 2.5 m) in the subcanopy, and a triple high net (30 × 7 m) in clearings, with a sampling intensity of 36,288 m^2^ nets/h, corresponding to 864 hr in 144 nights. The captured bats were handled according to the American Society of Mammalogists guidelines for the use of wild animals for research purposes (Sikes et al., 2016). After capture, the age, sex, and reproductive status were evaluated, and only adult males and adult nonpregnant, nonlactating females were used for measurements. Age was based on the degree of ossification of the wing joints. Reproductive status in females was determined by examining the nipples and palpation of the abdomen. Forearm length and body mass were recorded before euthanasia. Specimens were deposited in the biological collection of the University of Tolima CZUT‐M (Ibagué, Colombia), and skulls were cleaned for craniodental morphometry (Table S1).

The bite force of bats was measured using a portable digital fruit hardness tester Lutron FR 5120 (made in Taiwan) with a capacity of 196.10 Newton and precision ±0.05 that corresponded to in vivo measurements of the maximal force. Bite force was recorded at the molars, and measurements were repeated five times for each bat with a trial interval of at least 5 min following the method of Freeman and Lemen (2008). The maximum value of the five measurements was considered as the maximum bite force produced by that individual. The bite force of the species was calculated by averaging the maximum bite force of each individual.

**FIGURE 2:**
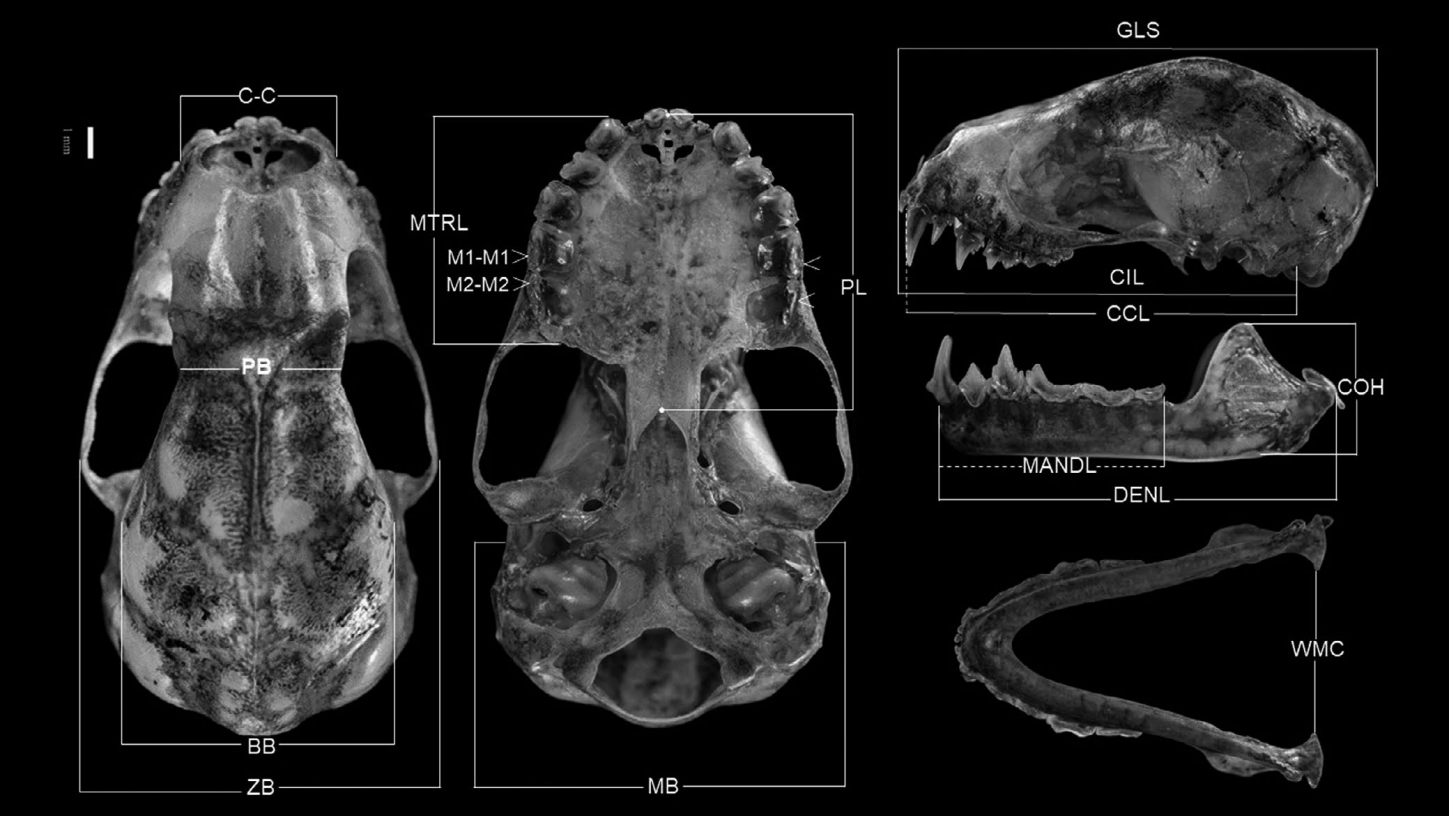
Lateral and dorsal views of the cranium and mandible (*Platyrrhinus helleri*, adult male), and ventral view of the cranium with measurements used in the craniodental morphometry of stenodermatine bats in Colombia. Abbreviations: GLS, greatest length of skull; CIL, condylo‐incisive length; CCL, condylo‐canine length; BB braincase breadth; ZB, zygomatic breadth; PB, postorbital breadth; C–C, palatal width at canines; MB, mastoid breadth; PL, palatal length; MTRL, maxillary toothrow length; M1–M1, width at M1; M2–M2, width at M2; DENL, dentary length; MANDL, mandibular toothrow length; COH, coronoid height; and WMC, width at mandibular condyles

The second procedure for collecting data consisted of measurements of 16 craniodental traits and two body traits (Table 1, Figure 2) from voucher specimens in the Zoological Collection of the University of Tolima (CZUT; Ibagué, Colombia), Museo Javeriano de Historia Natural “Lorenzo Uribe, SJ” (MPUJ; Bogotá, Colombia), and Royal Ontario Museum (ROM; Ontario, Canada). The specimens from these biological collections are from 24 localities in Colombia (Figure 1; Table S1). We verified that all were adult specimens based on the ossification of the phalange epiphyses in the wing (Dietz et al., 2007).

**TABLE 1:** The craniodental, mandibular, and external measurements used in this study of stenodermatine bats from Colombia

Variable	Main trait	Description	Abbreviation	Unit	Key supporting reference
Bite force	Head	Maximum bite force produced by molars	BF	N/g	Shi et al. (2020)
Forearm length	Body	Distance from the olecranon process to anterior surface of carpals in the folded wing	FA	mm	García‐Herrera et al. (2020)
Mass	Body	Weight of the body	MAS	g	Cisneros et al. (2014)
Greatest length of skull	Head	Distance from the posterior‐most point of the occiput to the anterior‐most point of the premaxilla, including incisors	GLS	mm	García‐Herrera et al. (2020)
Condylo‐incisive length	Head	Distance between a line connecting the posterior‐most margins of the occipital condyles and the anterior‐most surface of the upper incisors	CIL	mm	Cisneros et al. (2014)
Condylo‐canine length	Head	Distance between a line connecting the posterior‐most margins of the occipital condyles and a line connecting the anterior‐most surface of the upper canines.	CCL	mm	Cisneros et al. (2014)
Braincase breadth	Head	Breadth of the braincase, excluding mastoid and paraoccipital processes	BB	mm	Cisneros et al. (2014)
Zygomatic breadth	Head	Breadth across the zygomatic arches	ZB	mm	Cisneros et al. (2014)
Postorbital breadth	Head	Breadth at the postorbital constriction	PB	mm	Murillo‐García and De la Vega (2018)
Mastoid breadth	Head	Greatest breadth across the mastoid region	MB	mm	Murillo‐García and De la Vega (2018)
Palatal length	Head	Distance between the posterior palatal notch and the anterior border of the incisive alveolus	PL	mm	Murillo‐García and De la Vega (2018)
Maxillary toothrow length	Head	Distance from the anterior‐most surface of the upper canine to the posterior‐most surface of the crown of M3	MTRL	mm	Murillo‐García and De la Vega (2018)
Width at M1	Head	Greatest width of palate across M1s	M1‐M1	mm	Murillo‐García and De la Vega (2018)
Width at M2	Head	Greatest width of palate across M2s	M2‐M2	mm	Murillo‐García and De la Vega (2018)
Palatal width at canines	Head	Least width across palate between alveoli of upper canines	C‐C	mm	Murillo‐García and De la Vega (2018)
Dentary length	Head	Length between midpoint of condyle to anterior‐most point of dentary	DENL	mm	Murillo‐García and De la Vega (2018)
Mandibular toothrow length	Head	Distance from the anterior‐most surface of the lower canine to the posterior‐most surface of m3	MANDL	mm	Murillo‐García and De la Vega (2018)
Coronoid height	Head	Perpendicular height from ventral margin of mandible to tip of coronoid process	COH	mm	Murillo‐García and De la Vega (2018)
Width at mandibular condyles	Head	Greatest width between inner margins of mandibular condyles	WMC	mm	Murillo‐García and De la Vega (2018)

Among frugivorous bats, two foraging categories adapted to fruit consumption were distinguished based on Soriano (2000): nomadic frugivorous species whose strategy consists of feeding on trees with massive production of short‐lived fruit, while sedentary bats have search itineraries more or less fixed every night and focus the consumption of fruits in plants with continuous production throughout the year. This classification involves a specialization toward a group of plants, reducing competition and generating a coevolution with plants and bats (Fleming, 1986; Giannini & Kalko, 2004).

Stenodermatinae is the only subfamily with species that are either nomadic sedentary; therefore, these organisms constitute an excellent model to evaluate the skull traits involved in the consumption of contrasting fruits. We classified in our data set large‐sized nomadic species with weight 55–65 g and medium‐ to small‐sized nomadic with weight 10–18 g. Although our size limit is arbitrary, it may have some biological relevance because *Artibeus planirostris* and *A. lituratus* are large bats with high dispersal capacity (Trevelin et al., 2013).

Individuals captured in mist nets were placed in a clean cloth bag for up to 4 hr to obtain fecal samples, which were analyzed at a later date in the laboratory for seeds. The seeds were washed, examined with a dissection microscope, identified at the lowest possible taxonomic level (Table S2), and supplemented with bibliographic information from the registry (García‐Herrera et al., 2019b).

Fruits known as food were collected from Neotropical bats, and their hardness was recorded by a portable digital fruit hardness tester Lutron FR 5120. These measurements were made on 10 fruits taken in the field, and we averaged the values to estimate the hardness per fruit species. Fruits with hardness recorded as between 5 and 7 N were classified as hard, 4 N as intermediate, and 1 to 3 N as soft (see Table S3 for more details).

### 2.2 Statistical analyses

To analyze the intra‐ and interspecific morphological variation, the mean ± *SD* was calculated for all morphometric variables per species. The assumptions of normality and homoscedasticity were corroborated with Shapiro–Wilk's test and Levene's test, respectively. The preliminary analyses showed that our data fit a normal distribution and had homogeneity of variances, so we used a two‐way multivariate analysis of variance (MANOVA) to test for differences between species and sex. *Post hoc* comparisons between sexes were made within species to identify dimorphism.

A multiple linear regression was performed to evaluate the effects of body parameters on changes in bite force. In the models, the averages of bite force and body size (forearm length, greater skull length, and mass) of each species were used. The linear model was as follows: Ln (*y*) = *β*0 + *β*1*x*1 + *β*2*x*2 + *β*3*x*3 + *ε*, where *y* corresponds to the bite force (dependent variable); *x*1, *x*2, and *x*3 are the length of the forearm, the greatest length of the skull, and the mass, respectively (independent variables); *β*0 is the intercept; *β*1, *β*2, and *β*3 are the regression coefficients (for *x*1, *x*2, and *x*3, respectively); and *ε* is the random error term. A principal component analysis (PCA) of averages for species was used to visualize how they occupy morphospace. The correlation values showed a high association between the forearm length trait and mass (0.99; Table S4). Therefore, the rest of the morphometric features were used for the analysis canonical variate analysis (CVA) to establish the major axes of discrimination among individuals and between groups, to find the linear combinations of the initial variables with the maximum discriminating power between the groups, to test whether the means of these groups are significantly different, and to study the dimensionality of the data.

To determine the existence of significant variation between species and morphological traits, an analysis of covariance was performed using a generalized linear model (GLM). We use bite force as the response variable and cranial and body features as covariates (to correct for preexisting variation among species/sex). The model used was as follows: *Yijķ* = *µ* + *Ţi* + *δj* + Ω*ķ* + *εijķ*, where *Yijķ* represents the bite force response at the *j*‐th sex and the *i‐*th species; *µ* general average, *Ţi* effect produced by the *i*‐th species, *δj* effect produced by the *j*‐th sex, Ω*ķ* effect due to the *R*‐th trait, and *εijķ* the random error.

A multiple linear regression model was applied to study the relationship between bite force (*Y*) and the variables identified with the greatest influence in the previous models as explanatory variables. For the detection of masked variability, an intuitive and qualitative procedure based on graphic representation was used; then, post hoc tests were performed using Fisher's least significant difference (LSD) pairwise comparison procedure. Statistical significance for all tests was *p* ⩽ 0.05. All analyzes were performed in R 3.5.3 (R Core Team, 2019).

### 2.3 Phylogenetic signal

We tested the phylogenetic signal because it can create a confounding effect as taxa that are more closely related to exhibit similar trait values, and the similarity of traits decreases as the phylogenetic distance increases (Losos, 2008). For phylogenetic analyses, we used the phylogeny of Phyllostomidae corresponding to the maximum clade credibility tree used in Rolland et al. (2014). We trimmed the tree to only include the 40 frugivorous bats from Stenodermatinae. We estimated Blomberg's *K* with the function phylosignal, package picante (Blomberg et al., 2003; Kembel et al., 2010). Blomberg's *K* is the most appropriate metric because it is not significantly affected by polytomies, and is less sensitive to small sample sizes (20 taxa) than other indices (Münkemüller et al., 2012).

Values of *K*‐statistics lower than 1.0 indicate that related species resemble each other less than expected according to the Brownian motion model of trait evolution, while values greater than 1.0 mean that more related species are more similar, for the trait, under study, which was predicted by this model (Blomberg et al., 2003; Kamilar & Cooper, 2013).

### 2.4 Bite force measurements, cranial traits, and diet

Bite force was corrected for body size (forearm length, greater skull length, and mass), and the residuals were obtained. To find differences between species, the cranial features and bite force were used in a partial least square (PLS), technique that reduces the predictors to a smaller set of uncorrelated components and performs a least‐squares regression on these components, rather than using the original data (Vega‐Vilca & Guzmán, 2011). The PLS vector represents a linear vector in space that explains the maximum covariation between the cranial features and the residual bite force. Subsequently, we compared the variation in bite force, skull traits, and diet by means of a principal component analysis. The association between patterns of variation of skull traits in the PCA and restricted PLS will indicate whether the variation associated with bite force is associated with the main differences in interspecific skull traits and/or diet. The PLS analysis and the related correlation, permutation tests, and the PCA were performed in R 3.5.3 (R Core Team, 2019).

## 3 RESULTS

We studied the craniodental morphology of 343 specimens from seven species of stenodermatine bats in Colombia, of which 239 were collected from Tolima and used in the measurement of bite force: (a) nomadic frugivorous *Artibeus anderseni* (*n* = 30), *A. lituratus* (*n* = 35), *A. planirostris* (*n* = 76), *A. phaeotis* (*n* = 19), *Platyrrhinus helleri* (*n* = 22), and *Uroderma convexum* (*n* = 30) and (b) sedentary nomadic frugivorous *Sturnira giannae* (*n* = 27, Table S5). Comparisons of morphological traits showed significant differences between species (MANOVA; *F*_1,19_ = 86.87, *df* = 92, *p* < .001), sex (MANOVA; *F*_1,19_ = 1.25, *df* = 16, *p* < .05), and their interaction (MANOVA; *F*_1,19_ = 2.8, *df* = 94, *p* < .001; Table 2).

**TABLE 2:** Species, sex, and sample size (*n*) for bats used to investigate bite force and jaw‐skull shape relationships in Colombia

Species	Sex	*n*	Mass	FA	GLS	CIL	CCL	ZB	BB	PB	MB	PL	MTRL	M1‐M1	M2‐M2	C‐C	DENL	MANDL	COH	WMC	#	BS
*A. anderseni*	*♂*	22	10.70 (1.27)	36.64 (1.84)	18.26 (0.46)	13.98 (1.50)	13.9 (1.40)	10.56 (0.57)	8.87 (0.39)	4.60 (0.37)	8.73 (0.46)	7.6 (0.43)	5.38 (0.28)	7.74 (0.41)	7.39 (0.18)	4.29 (0.44)	10.9 (0.54)	5.90 (0.40)	4.42 (0.26)	5.92 (0.58)	16	35.50 (4.44)
	*♀*	22	10.77 (0.89)	36.16 (1.71)	18.11 (1.83)	14.21 (0.58)	14.12 (0.39)	10.71 (0.58)	8.88 (0.40)	4.54 (0.47)	9.11 (0.46)	7.54 (0.38)	5.41 (0.28)	7.59 (0.50)	7.49 (0.16)	4.15 (0.33)	10.93 (0.47)	5.87 (0.33)	4.52 (0.27)	5.68 (0.55)	14	65 (5.98)
*A. lituratus*	*♂*	39	58.47 (4.27)	68.81 (3.95)	30.69 (0.74)	24.35 (062)	23.76 (0.56)	18.34 (0.44)	14.12 (0.35)	6.58 (0.51)	14.58 (0.63)	14.63 (0.43)	10.51 (0.51)	12.78 (0.29)	12.74 (0.37)	6.41 (0.25)	20.41 (0.58)	12.65 (0.34)	9.64 (0.30)	9.58 (0.43)	18	65.44 (4.80)
	*♀*	37	62.10 (7.72)	69.18 (3.56)	31.34 (0.55)	25.06 (0.67)	24.44 (4.16)	18.62 (0.51)	13.93 (0.56)	7.12 (0.38)	14.72 (0.57)	15.13 (0.43)	11.1 (0.41)	12.77 (0.43)	12.87 (0.50)	6.33 (0.37)	20.68 (0.81)	12.59 (0.48)	9.56 (0.49)	9.87 (0.38)	17	77.68 (5.09)
*A. planirostris*	*♂*	40	58.29 (5.70)	59.95 (2.65)	27.87 (4.97)	22.58 (0.77)	22.07 (0.81)	16.78 (0.73)	13.32 (0.56)	6.63 (0.46)	12.63 (0.53)	13.54 (0.75)	10.41 (0.53)	11.46 (0.59)	10.65 (0.83)	5.74 (0.39)	19.26 (0.70)	11.15 (0.72)	7.89 (0.56)	8.42 (0.38)	38	76.02 (3.93)
	*♀*	40	55.84 (4.53)	59.86 (5.99)	28.12 (0.85)	22.71 (0.83)	22.21 (0.77)	17.19 (0.40)	13.47 (0.70)	6.98 (0.34)	13.19 (0.38)	13.88 (0.47)	10.61 (0.65)	11.53 (0.55)	11.50 (0.81)	6.28 (0.32)	19.32 (0.34)	11.49 (1.21)	8.48 (0.45)	8.56 (0.35)	38	93.25 (2.75)
*A. phaeotis*	*♂*	15	10.90 (0.99)	36.25 (1.25)	18.68 (0.64)	15.11 (0.78)	14.89 (1.00)	11.03 (0.48)	9.27 (0.47)	4.33 (0.32)	8.56 (0.55)	7.65 (0.77)	5.69 (0.55)	7.5 (0.51)	7.34 (0.40)	4.11 (0.25)	11.14 (0.57)	6.14 (0.64)	4.35 (0.43)	6.24 (0.59)	9	33.14 (1.76)
	*♀*	15	11.57 (1.92)	35.56 (1.80)	18.33 (0.77)	14.21 (0.58)	14.24 (0.52)	10.71 (0.41)	8.95 (0.39)	4.45 (0.21)	9.35 (0.42)	7.66 (0.68)	5.54 (0.42)	8.02 (0.52)	7.45 (0.21)	4.35 (0.33)	11.36 (0.55)	6.02 (0.51)	4.69 (0.25)	5.12 (0.85)	10	62.63 (2.20)
*P. helleri*	*♂*	14	16.93 (3.05)	39.78 (1.64)	21.25 (0.48)	17.58 (0.40)	17.21 (0.45)	10.44 (0.45)	8.58 (0.48)	4.89 (0.37)	8.81 (0.29)	9.73 (0.44)	7.55 (0.37)	7.92 (0.35)	7.69 (0.51)	3.85 (0.28)	13.65 (0.44)	8.32 (0.64)	4.96 (0.43)	5.96 (0.44)	11	44.73 (0.56)
	*♀*	20	17.93 (2.14)	41.04 (1.22)	21.88 (0.68)	17.99 (0.60)	17.33 (0.52)	11.16 (0.47)	9.5 (0.62)	5.24 (0.32)	9.15 (0.38)	9.99 (0.33)	7.72 (0.45)	8.02 (0.31)	8.12 (0.41)	3.84 (0.39)	13.74 (0.63)	8.64 (0.41)	4.60 (0.44)	6.13 (0.42)	11	72.89 (1.06)
*U. convexum*	*♂*	21	14.92 (1.35)	41.17 (0.87)	22.12 (0.65)	18.32 (0.67)	17.56 (0.49)	11.69 (0.52)	9.40 (0.36)	5.66 (0.65)	9.25 (0.41)	11.23 (0.56)	7.85 (0.43)	8.12 (0.62)	8.22 (0.44)	4.00 (0.53)	14.24 (0.28)	8.25 (0.49)	5.04 (0.55)	6.28 (0.27)	16	46.03 (0.97)
	*♀*	24	15.04 (1.21)	41.59 (0.78)	22.34 (0.44)	17.58 (0.70)	17.25 (0.54)	11.67 (0.68)	10.24 (0.48)	5.32 (0.47)	10.34 (0.70)	11.39 (0.43)	7.67 (0.42)	8.16 (0.43)	7.69 (0.49)	3.87 (0.45)	13.67 (0.43)	8.25 (0.47)	4.69 (0.44)	6.19 (0.62)	14	79.69 (0.72)
*S. giannae*	*♂*	16	20.02 (2.79)	41.02 (1.17)	22.25 (0.62)	17.81 (0.69)	17.22 (0.63)	12.09 (0.34)	10.45 (0.26)	6.08 (0.37)	10.38 (0.56)	10.25 (0.53)	6.52 (0.21)	7.58 (0.28)	7.64 (0.27)	5.30 (0.44)	13.99 (0.53)	7.60 (0.34)	5.25 (0.36)	7.22 (0.48)	14	35.04 (0.46)
	*♀*	17	20.73 (2.50)	41.29 (1.44)	22.11 (0.49)	17.65 (0.47)	17.22 (0.41)	12.26 (0.41)	10.47 (0.36)	6.25 (0.40)	10.25 (0.28)	10.24 (0.30)	6.55 (0.52)	7.59 (0.29)	7.58 (0.22)	5.16 (0.40)	14.01 (0.41)	7.86 (0.34)	5.47 (0.23)	7.25 (0.44)	13	59.19 (0.75)
MANOVA (Wilks' lambda)					**Value**		***F***	***df***	***p***													
	Species				0.00005		86.87	96	<.0001													
	Sex				0.93		1.25	16	.023													
	Species * Sex				0.39		2.8	94	<.001													

Note

All measurements are presented in millimeters (mean ± *SD*) except for BS which are presented in Newton and mass in grams. Abbreviations as in Table 1 and Figure 2. The # symbol that precedes BS indicates the number of individuals that were used for bite force. The values inside the parentheses correspond to ±*SD*.

**FIGURE 3:**
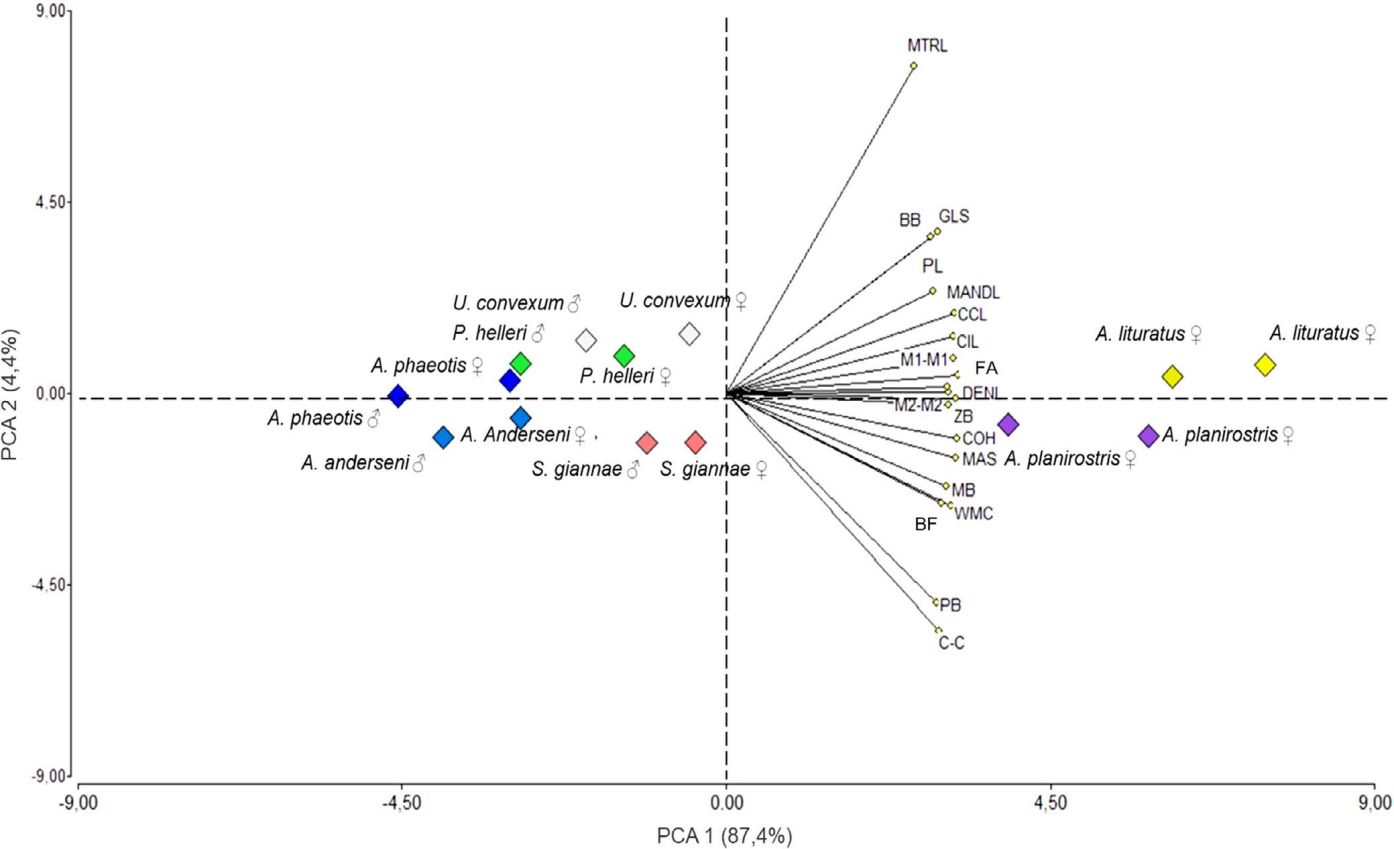
Distribution of body and craniodental traits for seven species of stenodermatine fruit bats from Colombia based on a principal components analysis (PCA)

The PCA showed that the first two principal components (PC) explained 91.8% of total variation (PC1 87.4% and PC2 4.4%, respectively). All variables had high positive eigenvectors on the first component indicating overall size (Table S6). The variables that were highly correlated with PC1 were forearm length (FA), mass (MAS), dentary length (DENL), coronoid height (COH), mandibular toothrow length (MANDL), condylo‐incisor length (CIL), and condyle‐canine length (CCL). PC2 showed the highest positive correlation with maxillary toothrow length (MTRL), but high negative values for width between the cingulate of the upper canines (C‐C) and postorbital breadth (PB) indicate a shorter broader rostrum. The species are clearly grouped according to size, on the positive side the large species *A. lituratus* and *A. planirostris* and on the left side the rest of the species (Figure 3).

**FIGURE 4:**
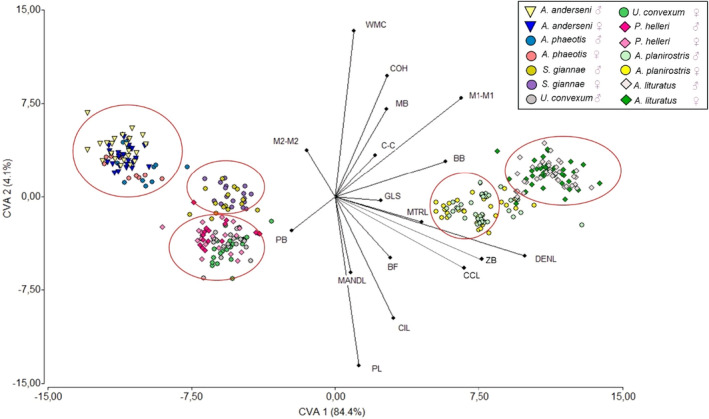
Canonical variate analysis with the description of body and craniodental traits of seven species of stenodermatine bats from Colombia. The ellipses highlight the five subgroups

According to the CVA, the traits that contributed most positively to axis1 were WMC, COH and M2‐M2, and negatively PL, MANDL, CIL and in CVA2 the traits GLS, MTRL and BB (Figure 4 and Table S5).

**FIGURE 5:**
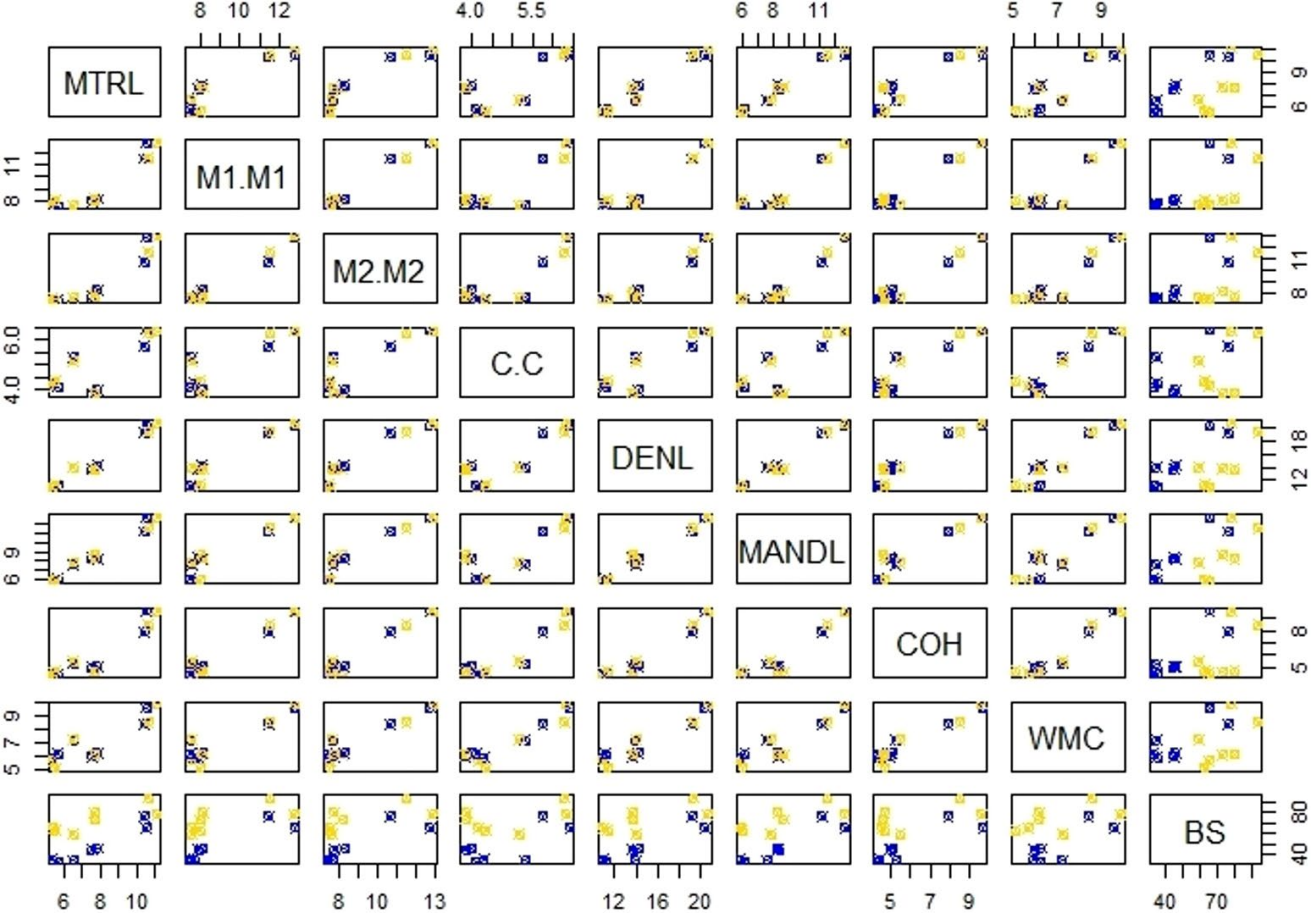
Pairwise relationships most significant of craniodental traits with bite force for seven Colombian species of stenodermatine bats. Blue squares correspond to males and yellow to females. Abbreviations as in Table 1

### 3.1 Variation in the bite force associated with craniodental traits

The GLM found that the bite force of stenodermatine bats was significantly influenced by all the cranial features (*p* < .001; Table 3). It was identified by multiple comparison analysis that 15 traits are significantly associated with bite force, and the traits with the highest correlation are MTRL, MANDL, M1‐M1, and DENDL regardless of sex (Figure 5, Table S7).

**TABLE 3:** Significance between pairs of species and relationship of bite force with other traits in stenodermatine fruit bats from Colombia

Trait	*p*‐value	Species	*p*‐value
Bf ‐ C‐C	<.001	*A. lituratus ‐ A. phaeotis*	<.001
Bf ‐ CCL	<.001	*A. lituratus ‐ A. anderseni*	<.001
Bf ‐ CIL	<.001	*A. lituratus ‐ A. planirostris*	.486
Bf ‐ COH	<.001	*A. lituratus ‐ P. helleri*	<.001
Bf ‐ DENL	<.001	*A. lituratus ‐ S. giannae*	<.001
Bf ‐ GLS	<.001	*A. lituratus ‐ U. convexum*	<.001
Bf ‐ M1‐M1	<.001	*A. phaeotis ‐ A. planirostris*	<.001
Bf ‐ M2‐M2	<.001	*A. phaeotis ‐ A. anderseni*	.84
Bf ‐ MANDL	<.001	*A. phaeotis ‐ P. helleri*	.13
Bf ‐ MASA	<.001	*A. phaeotis ‐ S. giannae*	.04
Bf ‐ MB	<.001	*A. phaeotis ‐ U. convexum*	.05
Bf ‐ MTRL	<.001	*A. planirostris ‐ A. anderseni*	<.001
Bf ‐ PB	<.001	*A. planirostris ‐ P. helleri*	<.001
Bf ‐ PL	<.001	*A. planirostris ‐ S. giannae*	<.001
Bf ‐ WMC	<.001	*A. planirostris ‐ U. convexum*	<.001
Bf ‐ ZB	<.001	*A. anderseni ‐ P. helleri*	.08
		*A. anderseni ‐ S. giannae*	.08
		*A. anderseni ‐ U. convexum*	.04
		*P. helleri ‐ S. giannae*	.99
		*P. helleri ‐ U. convexum*	.73
		*S. giannae ‐U. convexum*	.73

Grey shade indicates significant vlaues *p* > .001.

### 3.2 Interspecific bite force variation

**FIGURE 6:**
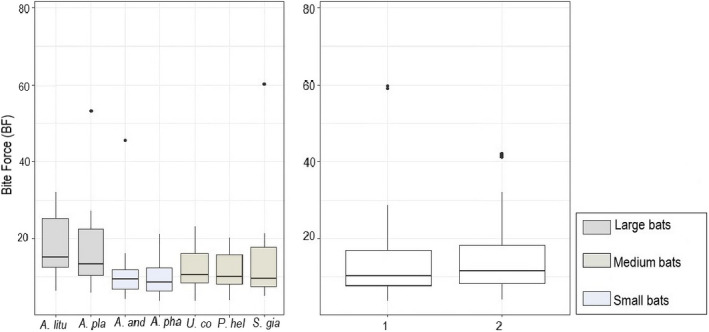
Box plot showing differences in relative bite force (median) among bat species and sex. Outlier values are also indicated. *A. lit*: *Artibeus lituratus. A. pla*: *A. planirostris. A. and*: *A. anderseni. A. pha*: *A. phaeotis. U. con: Uroderma convexum*. *P. hel*: *Platyrrhinus helleri* and *S gia: Sturnira giannae*

The bite force varied between species; large bats (*A. lituratus* and *A. planirostris*) had the highest bite force, followed by medium‐sized bats (*P. helleri, U. convexum*, and *S. giannae*). The lowest force was recorded for the small‐sized bats of the species *A. anderseni* and *A. phaeotis* (*p* < .001). Significant intersexual difference between species was detected with females having a greater bite force compared with males (*p* < .04; Figure 6).

Although the analysis of variation of the interspecific bite force showed that the bite force is directly proportional to the size of the bat, the results of the GML determined that the bite force varies differently from the size. Obviously, the large species showed significant differences in bite force in relation to the other species. However, for the couples who presented *p* > .05, it is accepted that the null hypothesis is true; therefore, the bite force of *A. phaeotis* is similar to that of *P. helleri*, but greater than that of *S. giannae* and *U. convexum,* while *A. anderseni* registered a bite force similar to that of *P. helleri* and *S. giannae*, but greater than that of *U. convexum* (Table 3).

### 3.3 Differences in bite force between bat species

Significant differences in skull morphology and bite strength were observed between species (Table 3). LSD post hoc testing clustered species into 4 groups: The first group consisted of the large‐sized bats, *A. lituratus* and *A. planirostris*; the second group included the medium‐sized species, *U. convexum*; the third group formed by the medium and small species, *P. helleri*, *S. giannae*, and *A. phaeotis*; and the fourth group formed by the small species, *A. anderseni* (Table 4).

**TABLE 4:** Fisher's least significant difference (LSD) pairwise comparison of body/cranial traits in stenodermatine fruit bat species from Colombia

Species	Group
*A. lituratus*	a
*A. planirostris*	a
*U. convexum*	b
*P. helleri*	bc
*S. giannae*	bc
*A. phaeotis*	bc
*A. anderseni*	c

### 3.4 Phylogenetic signal

Traits had a *K*‐value between zero and unity; only the CIL, CCL, MTRL had *K* > 1, indicating that this trait is phylogenetically conserved. While the features of the cranium, greatest length of skull (GLS), braincase breadth (BB), zygomatic breadth (ZB), palatal length (PL), and de la mandible, dentary length (DENL), mandibular toothrow length (MANDL), and coronoid height (COH) presented a had a significant *K* < 1. significant result according to the p‐value (Table 5). Evidenced as a result that these traits have evolved independently of the phylogeny.

**TABLE 5:** *K*‐statistics resulted from Blomberg's tests for phylogenetic signal and its respective *p*‐values are provided for every trait

Trait	K	Se. Dev.	*p*‐value
Cranium			
GLS	0.89	0.58	.001
CIL	1.24	−1.21	.356
CCL	1.04	2.74	.442
BB	0.94	0.75	.001
ZB	0.75	−0.58	.001
PB	0.85	−1.45	.812
C‐C	0.98	1.14	.756
MB	0.97	−0.14	.291
PL	0.98	1.16	.001
MTRL	1.24	1.98	.001
M1‐M1	0.78	0.58	.812
M2‐M2	0.72	1.18	.684
Mandible			
DENL	0.82	−1.36	.038
MANDL	0.68	0.11	.01
COH	0.75	0.25	.01
WMC	0.94	0.39	.854

Grey shade indicates significant vlaues *p* < .05.

### 3.5 Bite force measurements, cranial traits, and diet

**FIGURE 7:**
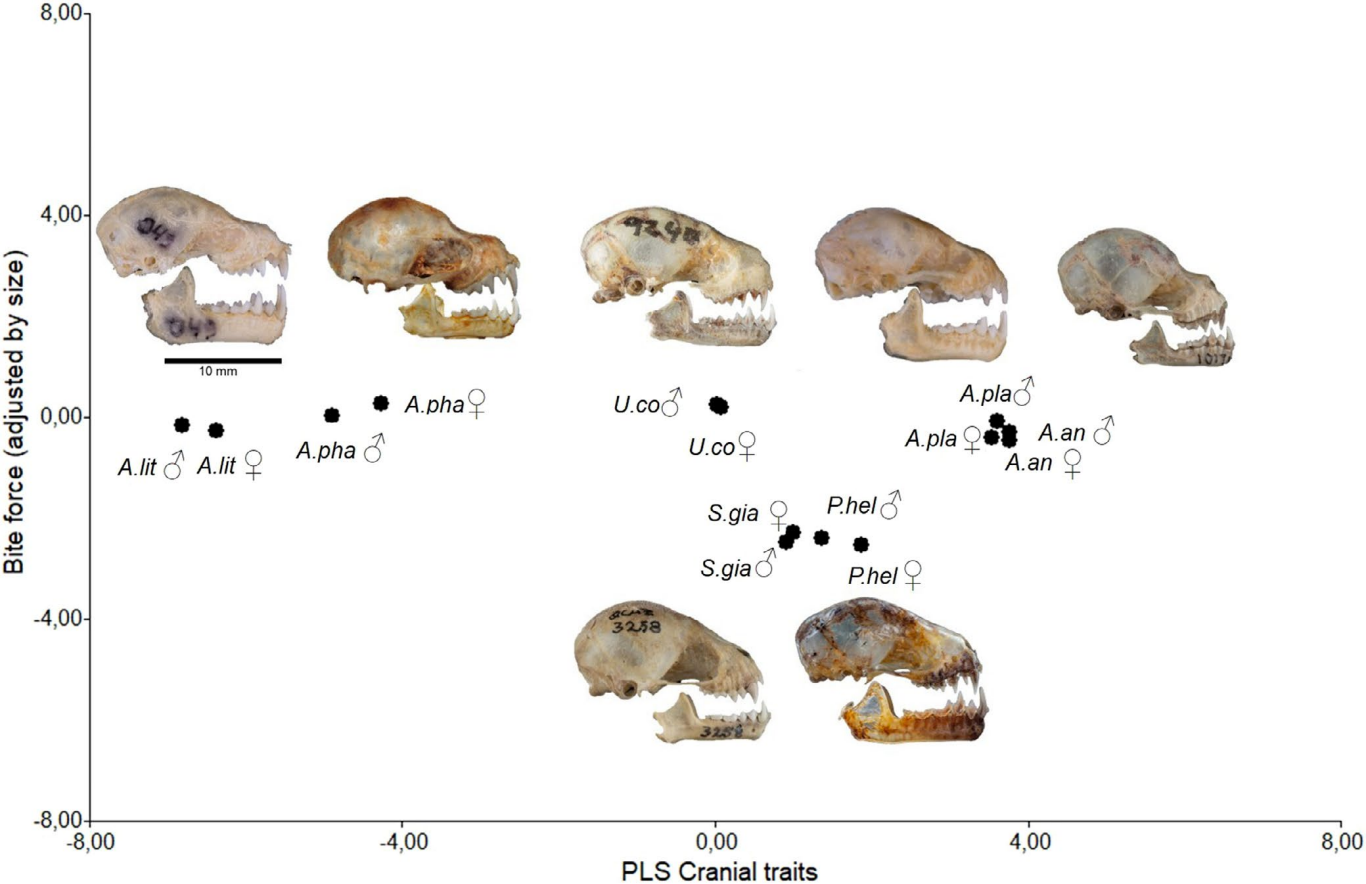
Regression of size‐adjusted bite force against the partial least‐squares (PLS) analysis skull traits vector. *A.an: Artibeus anderseni, A.lit: A. lituratus, A.pha: A. phaeotis, A.pla: A. planirostris, P.hel: Platyrrhinus helleri, U.co: Uroderma convexum,* and *S.gi: Sturnira giannae*

Our PLS result with size‐corrected bite force reveals that residual bite force differs indistinctly from size, for example, large *A. lituratus* and small *A. phaeotis* groups on negative side of the plot, whereas large *A. planirostris* and small *A. anderseni* groups on the positive side (Figure 7).

**FIGURE 8:**
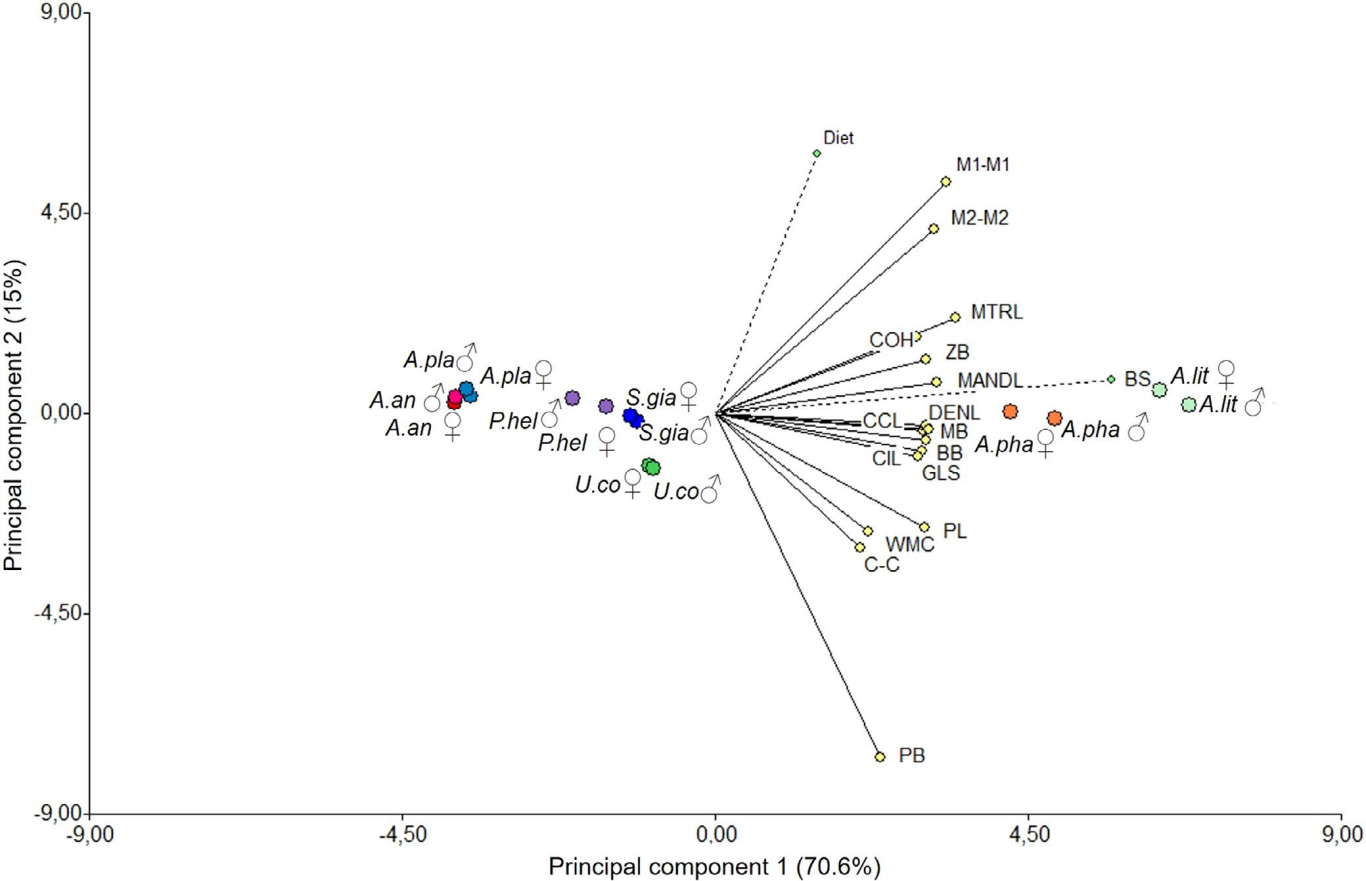
Principal component analysis biplot for stenodermatine bats diet scores in Colombia. The diet was registered according to the fruit's ripeness (see Tables S2 and S3). Species scores are represented by italicized species acronyms. Diet variables are represented as vectors identified by the variable names in bold. Abbreviations as in Figure 2

The first CP of the association between bite force, cranial shape, and diet explains 70.6% of the total variation and shows that DENL, MANDL, BB, MB, GLS, CIL, and CCL are associated with bite force. The second CP explains 15% and is negatively associated with the PB and positively with diet, M1‐M1 and M2‐M2. High bite force values are associated with the DENL and MANDL, traits that could explain the better performance of the *A. lituratus* and *A. phaeotis* species with respect to the other species of the same genus studied here. Although *A. anderseni* and *A. planirostris* also had high bite forces, this was associated with diet, which could indicate resource‐dependent modulation of bite force. Diet was also a determining factor for bite force for *U. convexum*, *S. giannae,* and *P. helleri* species, but skull characteristics were not associated with residual bite force, linking diet with a weaker bite. The associations of the species with diet and skull traits were regardless of sex (Figure 8).

## 4 DISCUSSION

Neotropical leaf‐nosed bats (Phyllostomidae) are an ecologically diverse group of mammals with distinctive morphological adaptations associated with specialized modes of feeding (Camacho et al., 2019). In particular, Stenodermatinae have a strict nomadic frugivorous diet, related to the consumption of hard and soft fruits (Dumont et al., 2012), which is linked to a strong bite that is associated with a short and wide skull (Santana et al., 2010, 2012). Fruit feeders are morphologically diverse, exhibiting cranial and mandibular morphologies that overlap with other guilds (Dumont et al., 2012). This morphological diversity reflects the variety of physical properties represented among fruits and the tendency of frugivorous species to specialize in particular fruits (Rossoni et al., 2017; Santana et al., 2010). These specializations are determined by the functional traits that allow them to exploit different fruits.

In our study, we found differences in skull, forearm, and mass variables between males and females in the seven species of stenodermatines studied here. This variation may be associated with the diet of bat species, possibly as a result of differences in energy requirements during the reproductive season (de Camargo & de Oliveira, 2012). Although sexual dimorphism has been relatively well documented in evening bats (family Vespertilionidae), with females larger and heavier than males (Bornholdt et al., 2008), in phyllostomid bats this information is less documented despite being the most diverse family and distributed in the Neotropics (Gardner, 2008). One exception is López‐Aguirre and Pérez‐Torres (2015) identified that *Artibeus lituratus* females in Colombia had greater fluctuating asymmetry in the splanchnocranium, resulting in a differential bite force between the sexes.

Bite force has been established as an important performance trait for vertebrates that is associated with both cranial morphology and trophic ecology (Santana et al., 2010). Our study shows, for the first time, strong quantitative evidence of such a correlation by using a dataset of cranial variables involved in bite force within a morphologically diverse clade of New World fruit bats. The Stenodermatinae subfamily are morphologically diverse with cranial and mandibular features that overlap with other feeding guilds (Santana et al., 2012), which is reflected in the variety of foods, as well as in the tendency of these species to specialize in a group of particular fruits (Rojas et al., 2012; Rossoni et al., 2017). Several craniodental features contribute to generating a greater bite force, and the differences between these particular features have shown a clear segregation between species (Santana et al., 2012).

The association between craniodental features and bite force by species can be explained by the type of fruit consumed. For example, *A. phaeotis*, *P. helleri,* and *S. giannae* formed an independent group in the least significant difference pairwise comparison test. These species have a short skull and a shorter distance from the teeth to the jaw joint, which allows them to consume hard and soft fruits (Arias & Pacheco, 2019; Dumont et al., 2012; García‐Herrera et al., 2019b; Santana et al., 2012). *A. anderseni* and *U. convexum* each formed an identical group, but they share skull features with species of the previous group. *A. anderseni* can easily be confused with *A. phaeotis* based on the morphology of the skull; however, the rostrum is usually elevated anteriorly (versus. straight and palatal length shorter than the postpalatal length in *A. phaeotis* Díaz et al., 2016), while *U. convexum* presents a parallel rostrum and a short face that abruptly expands from the front to the edges of the lacrimal bone (Mantilla‐Meluk, 2014). These characteristics allow them to exploit mainly hard fruits of plant species such as *Ficus* spp. (Sagot & Stevens, 2012). The large species *A. lituratus* and *A. planirostris* are readily distinguished by size from the other fruit‐eating bats.

Our study did not consider the phylogenetic correction of the species; therefore, it presents limitations to understand how evolutionary changes in the diet of bats are correlated with skull traits, and how they modulate bite force. Clearly, our understanding focuses on morphological analyzes and on the low phylogenetic signal found among the traits studied here for Stenodermatinae, concluding that the changes presented in bite force are modulated by greatest length of skull, braincase breadth, zygomatic breadth, palatal length, and de la mandible; dentary length, mandibular toothrow length, and coronoid height. Traits that have diverged as a result of environmental pressures (Pitnick et al., 2006; Santana et al., 2012). Murillo‐García (2018) found several adaptive changes across the phylogeny of neotropical fruit bats (Phyllostomidae: Stenodermatinae and Carollinae), indicating divergence in skull and jaw morphology. Stenodermatins have the additional ability to consume both soft and hard fruits (Aguirre et al., 2003; Dumont et al., 2009). Therefore, the range of dietary niches available for stenodermatine bats is indeed broader than that of other phylogenies, which possibly determined that the traits varied according to aspects other than closeness in phylogeny (Diniz‐Filho et al., 2012). Previous work has shown a significant change in the diversification rates at the base of Stenodermatinae (Jones et al., 2005; Shi & Rabosky, 2015), which we confirm is reflected in the morphology from a morphometric‐based perspective.

The traits that indicated that they have evolved independently of the phylogeny, were also the traits associated with bite force; dentary length, mandibular toothrow length, coronoid height, braincase breadth, condyle‐ canine length, condyle‐incisive length, and greatest length of skull. Furthermore, these traits were directly related to the diet of *A. phaeotis* and *A. lituratus*.

We show that an additional part of the variation in bite force can be attributed to differences in skull traits, foraging strategy, and diet. For example, a short rostrum, together with a greater dentary length, mandibular toothrow length, and a high coronoid, increases the residual bite force when size is accounted for. In this way, the smaller nomadic species such as *A. anderseni* and *A. phaeotis* have a bite force similar to the large‐sized nomadic species *A. planirostris* and *A. lituratus*, respectively. By contrast, *P. helleri* (medium‐sized nomadic species) register a bite force similar to the sedentary bat, *S. giannae*. Species that presents a globular cranial box, with zygomatic arches that do not converge anteriorly (Velazco & Patterson, 2019), which possibly allows it to have a bite force similar to robust skulls and a wide rostrum, present in *P. helleri* and *U. convexum*.

**FIGURE 9:**
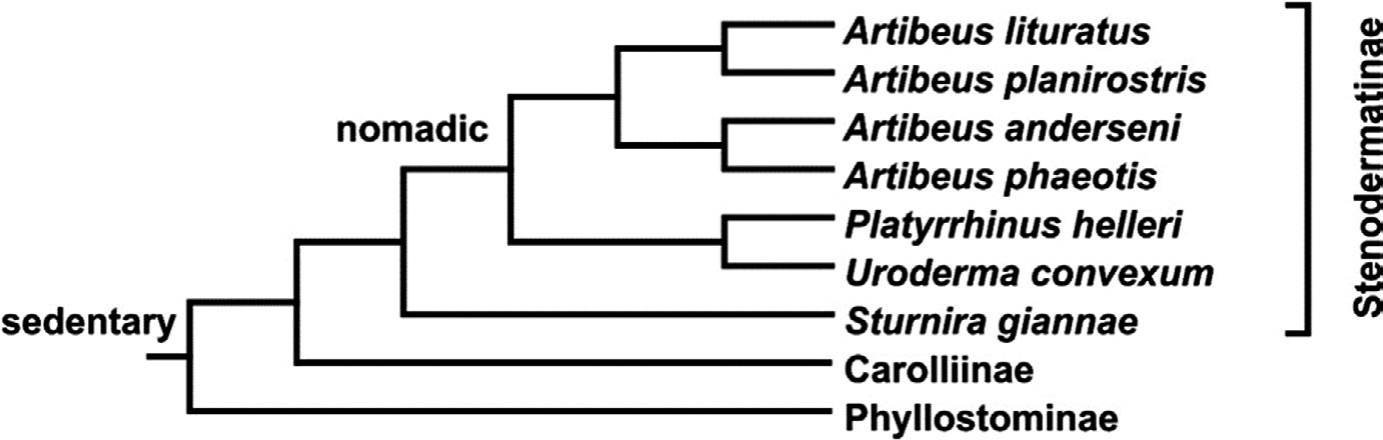
Phylogeny of stenodermatine fruit bats used in our study rooted with two other subfamilies of Phyllostomidae. Sedentary and nomadic foraging strategies are parsimoniously mapped onto the tree based on Soriano (2000) and Rolland et al. (2014)

There is a strong and positive correlation between the mass (size) of the food and the maximum force required to eat food, showing that hard fruit (e.g., figs) requires more force than soft fruit (e.g., *Solanum*), such that larger bats can process larger and harder foods (Aguirre et al., 2003; Arbour et al., 2019; Santana et al., 2012). Given that nomadic bats can consume some similar fruits, we expected that bats grouped in this category would have similar bite forces. However, our results established that the nomadic species *P. helleri* and *U. convexum* have a similar residual bite force to the sedentary species *S. giannae*, possibly because these bats with differing foraging strategies feed on food resources that have different mechanical demands when consuming fruits or other resources. However, this similarity in bite force could have a phylogenetic explanation. The frugivorous foraging strategies in Phyllostomidae based on Soriano (2000) and Rolland et al. (2014) suggest that sedentary feeding is the ancestral behavior for the family and nomadic feeding in Stenodermatinae is a derived behavior (Figure 9). This indicates that there is a phylogenetic basis to foraging in Neotropical fruit‐eating bats.

Our study shows strong quantitative evidence of the relationship between diet, foraging strategy, skull traits, and bite force in stenodermatine bats. Our results, however, are limited by the number of species included in the analysis. This was a consequence of the difficulty in catching many of the potentially rare frugivorous species in tropical dry forest of Colombia. Additionally, information on bite force available for phyllostomids bats in the literature is limited. Future studies should focus on collecting bite force data for other species of bats to get a better understanding of functional morphological variation.

Our findings illustrate that skull size is a determining factor in the bite force, but emphasizes that the use of functional traits is relevant for establishing the feeding performance of bat species. This highlights the importance of studying the relationships between morphology, bite force, ecology, and phylogeny of the species to get a better understanding of evolutionary adaptions of highly diverse Neotropical bat groups.

## CONFLICT OF INTEREST

None declared.

## AUTHOR CONTRIBUTIONS

**Leidy Viviana García‐Herrera:** Conceptualization (equal); data curation (equal); formal analysis (equal); funding acquisition (equal); investigation (equal); methodology (equal); visualization (lead); writing—original draft (equal); writing—review & editing (equal). **Leidy Azucena Ramírez‐Fráncel:** Data curation (equal); formal analysis (equal); investigation (supporting); methodology (supporting); writing—original draft (equal); writing—review & editing (equal). **Giovany Guevara:** Conceptualization (supporting); data curation (supporting); formal analysis (equal); supervision (supporting); validation (equal); writing—review & editing (supporting). **Gladys Reinoso‐Flórez:** Funding acquisition (equal); investigation (equal); supervision (equal); validation (supporting); writing—original draft (supporting). **Alfonso Sánchez‐Hernández:** Data curation (equal); formal analysis (lead); methodology (equal). **Burton K. Lim:** Conceptualization (lead); supervision (equal); writing—review & editing (lead). **Sergio Losada‐Prado:** Conceptualization (equal); investigation (equal); project administration (equal); supervision (lead); validation (lead); visualization (equal); writing—original draft (equal); writing—review & editing (equal).

### OPEN RESEARCH BADGES

This article has earned an Open Data, Open Materials and Preregistered for making publicly available the digitally‐shareable data necessary to reproduce the reported results. The data is available at https://datadryad.org/stash/share/N6vW4UGTQZbdh132I0gaPR2GYsa4iDCy5YdsEeNaerM.

## Supporting information

Table S1‐S7

## Data Availability

The data are available at https://datadryad.org/stash/share/N6vW4UGTQZbdh132I0gaPR2GYsa4iDCy5YdsEeNaerM.

## APPENDIX A

### Specimens examined

The following list includes all specimens examined in this study of stenodermatine fruit bats from Colombia, with their respective localities. Abbreviations for museum collections are given in the Materials and Methods.

*Artibeus anderseni* (*n* = 44)—AMAZONAS, Leticia (ROM 53614, 53615, 53616, 63061) ANTIOQUIA, Los Remedios (ROM 84982), CUNDINAMARCA, La Gran Curva, 114 km al Oeste de Bogotá (ROM 48959, 51790), Melgar (ROM 53613, 75300, 75302), Puerto Salgar (ROM 44956, 44957, 44965), TOLIMA, Alvarado, vereda Rincón de Chipalo, Parque Nacional del Arroz (CZUT‐M 2089, 2090, 2091, 2099, 2100, 2125, 2126, 2170), Armero Guayabal, Centro Universitario Regional del Norte—Universidad del Tolima (CZUT‐M 1128, 1415, 1416, 1418, 1434, 1436, 1481, 1492, 1515, 1516, 1517, 1536, 1537, 1538, 1541, 1624, 1638, 1639, 1641, 1642), Espinal (ROM 88089), Ibagué, vereda Aparco (CZUT‐M 2098, 2103).

*Artibeus lituratus* (*n* = 76)—CAUCA, Bellavista (ROM 63232, 63063, 64066, 64067, 64068, 64069, 64070, 64071), Munchique (ROM 67247, 67253, 67255, 67256, 67261), CUNDINAMARCA, Puerto Salgar (ROM 44811), META, Villavicencio, Puerto López (ROM 88083), TOLIMA, Alvarado, vereda Rincón de Chipalo, Parque Nacional del Arroz (CZUT‐M 2130, 2134, 2138, 2150), Ambalema, vereda Chorrillo (CZUT‐M 1410, 1546, 1567, 1569, 1668), Armero Guayabal, Centro Universitario Regional del Norte—Universidad del Tolima (CZUT‐M 1124, 1413, 1545, 1546, 1564, 1565, 1566, 1596, 1628, 1629, 1631, 1704, 2121, 2122, 2136, 2137), Ibagué, vereda Aparco (CZUT‐M 2129, 2132, 2133), Chucuni (CZUT‐M 0763, 0764, 0766, 0835), Buenos Aires (ROM 44882, 44885), Melgar, Santo Tomas (MUJ 00826, 00827, 00828, 00829, 00830, 00832, 00833, 00834) Suárez, vereda Batatas (CZUT‐M 0299, 0301, 0303, 0305, 0344, 0346, 2131), vereda Aguas Claras (CZUT‐M 0363), vereda Los Arrayanes (CZUT‐M 1230, 1231), PUTUMAYO, vereda Guascayaco (ROM 46356, 49206, 49209, 49211), VAUPÉS, Mitú (ROM 45258, 45259, 45260), VALLE DEL CAUCA (ROM 44889, 44890).

*Artibeus planirostris* (*n* = 80)—META, Fuente de Oro, Km 9 Carretera Puerto Limón (ROM 90109, 91409), TOLIMA, Ambalema, vereda Chorrillo (CZUT‐M 1468, 1469, 1470, 1471, 1472, 1473, 1474, 1475, 1476, 1477, 1494, 1544, 1568, 1583, 1584, 1585, 1586, 1587, 1588, 1589, 1590, 1591, 1592, 1599, 1600, 1605, 1632, 1643, 1644, 1645, 1646, 1669, 1670, 1671, 1672, 1673, 1695, 1696, 1697, 1699, 1700, 1701), Ibagué, vereda Aparco (CZUT‐M 1994, 1995, 1996), Armero Guayabal, Centro Universitario Regional del Norte—Universidad del Tolima (CZUT‐M 1412, 1414, 1493, 1513, 1514, 1542, 1543, 1593, 1594, 1595, 1598, 1698, 1705, 1712, 2008, 2049, 2139, 2140, 2143, 2165), Suárez, vereda Batatas (CZUT‐M 0243, 0244, 0245, 0298, 0300, 0302, 0304, 0345, 0347, 2070, 2164), PUTUMAYO, vereda Guayaco (ROM 49211), vereda San Miguel (ROM 67246).

*Artibeus phaeotis* (*n* = 30)—AMAZONAS, Leticia (ROM 53610, 53611, 53612), CAUCA, Bellavista (ROM 64064, 64059, 64057), MAGDALENA, Santa Marta (ROM 79885), TOLIMA, Alvarado, vereda Rincón de Chipalo, Parque Nacional del Arroz (CZUT‐M 2106), Ambalema, vereda Chorrillo (CZUT‐M 1346, 1549, 1572, 1647) Armero Guayabal, Centro Universitario Regional del Norte—Universidad del Tolima (CZUT‐M 1127, 1435, 1437, 1518, 1571, 1625, 1640, 1702, 1703, 1706, 1710, 1711, 2166), San Sebastián de Mariquita (MUJ 0269, 0285, 0286), Melgar (MUJ 00835), Suárez, vereda Batatas (CZUT‐M 2163).

*Platyrrhinus helleri* (*n* = 35)—MAGDALENA, Santa Marta (ROM 79882), TOLIMA, Alvarado, vereda Rincón de Chipalo, Parque Nacional del Arroz (CZUT‐M 2009, 2062, 2169, 2182), Ambalema, vereda Chorrillo (CZUT‐M 1709), Armero Guayabal, Centro Universitario Regional del Norte—Universidad del Tolima (CZUT‐M 1539, 1547, 1570, 1602, 1626, 1630, 2010), Líbano, Hacienda La Trinidad (ROM 88082), Ibagué, vereda Aparco (CZUT‐M 2160, 2161), Martínez (CZUT‐M 0027, 0116), San Sebastián de Mariquita (CZUT‐M 1063, 1065, 1072), Melgar (CZUT‐M 1928), vereda Santo Tomas (MUJ 00837, 00838, 00839, 00841), Suárez, vereda Batatas (CZUT‐M 0341, 0364, 1914), PUTUMAYO (ROM 403559, 46375, 46353, 63239), VAUPÉS, Mitú (ROM 45274, 45273).

*Uroderma convexum* (*n* = 45)—MAGDALENA, Santa Marta (ROM 79886), TOLIMA, Alvarado, vereda Rincón de Chipalo, Parque Nacional del Arroz (CZUT‐M 2093, 2101, 2104, 2105, 2158, 2159, 2171, 2183, 2195), Armero Guayabal, Centro Universitario Regional del Norte—Universidad del Tolima (CZUT‐M 1126, 1438, 1511, 1512, 1540, 1597, 1601, 1622, 1623, 1633, 1634, 1635, 1636, 1637, 1694, 1707), Ibagué, vereda Aparco (CZUT‐M 2095, 2096, 2097, 2102), San Sebastián de Mariquita (CZUT‐M 1054, 1070), Melgar (CZUT‐M 1927, MUJ 00842, 00843, 00844, ROM 62508), Suárez, vereda Batatas (CZUT‐M 1915), PUTUMAYO, vereda Guascayaco (ROM 46360), vereda Horno (ROM 63240, 63240), VAUPÉS, Mitú (ROM 45268, 45267, 45366, 45265).

*Sturnira giannae* (*n* = 33)—TOLIMA, Alvarado, vereda Rincón de Chipalo, Parque Nacional del Arroz (CZUT‐M 2146, 2147, 2172), Ambalema, vereda Chorrillo (CZUT‐M 1116, 1117, 1118, 1296, 1310, 1318, 1342, 1343), Armero Guayabal, Centro Universitario Regional del Norte—Universidad del Tolima (CZUT‐M 1129, 1403), Ibagué, vereda Aparco (CZUT‐M 2014, 2015, 2016, 2021, 2022, 2023, 2028), San Sebastián de Mariquita (CZUT‐M 1056, 1061), Suárez, vereda Batatas (CZUT‐M 0234, 0246, 0307, 0362, 1236), PUTUMAYO (ROM 40374, 40313, 40349, 40375, 46373, 49184).
